# Development of Integrative Methodologies for Effective Excavation Progress Monitoring

**DOI:** 10.3390/s21020364

**Published:** 2021-01-07

**Authors:** Abdullah Rasul, Jaho Seo, Amir Khajepour

**Affiliations:** 1 Department of Automotive and Mechatronics Engineering, Ontario Tech University, Oshawa, ON L1G 0C5, Canada; abdullah.rasul@ontariotechu.net; 2 Department of Mechanical and Mechatronics Engineering, University of Waterloo, Waterloo, ON N2L 3G1, Canada; a.khajepour@uwaterloo.ca

**Keywords:** excavation progress, ground volume estimation, bucket volume estimation, occlusion area, proprioceptive and exteroceptive sensors, 5D mapping, stereo vision camera, LiDAR, convolutional neural network

## Abstract

Excavation is one of the primary projects in the construction industry. Introducing various technologies for full automation of the excavation can be a solution to improve sensing and productivity that are the ongoing issues in this area. This paper covers three aspects of effective excavation progress monitoring that include excavation volume estimation, occlusion area detection, and 5D mapping. The excavation volume estimation component enables estimating the bucket volume and ground excavation volume. To achieve mapping of the hidden or occluded ground areas, integration of proprioceptive and exteroceptive sensing data was adopted. Finally, we proposed the idea of 5D mapping that provides the info of the excavated ground in terms of geometric space and material type/properties using a 3D ground map with LiDAR intensity and a ground resistive index. Through experimental validations with a mini excavator, the accuracy of the two different volume estimation methods was compared. Finally, a reconstructed map for occlusion areas and a 5D map were created using the bucket tip’s trajectory and multiple sensory data with convolutional neural network techniques, respectively. The created 5D map would allow for the provision of extended ground information beyond a normal 3D ground map, which is indispensable to progress monitoring and control of autonomous excavation.

## 1. Introduction

An excavator is one of the most significant pieces of equipment in the construction industry, and is used for earthworks since they can perform various tasks such as loading, leveling, cutting, and grading earth [1]. During the operation of excavation, operators use their sense together with reasoning-based knowledge and experience to monitor and control the excavation process. However, this area has been struggling to find skilled equipment operators due to recent labor shortages, and thus the transition from manual to autonomous excavation can be a solution to this problem.

Effective and accurate monitoring of the excavation process is an important component for autonomous excavation since it provides a foundation for successful operation control and process planning/management. To facilitate excavation progress monitoring, various approaches for excavation ground detection and mapping have been developed as follows.

To estimate the ground volume, Zhang et al. [2] applied and compared two methodologies for volume estimation based on laser scanner measurements. In this study, the first method was used to calculate the volume with 3D models or meshed surfaces whereas the second method allowed the same task using the isolated points from measurements whose single element (point) is considered as an apex of the truncated pyramid in 3D space. However, the study showed the limited accuracy of the estimated volume due to under-sampling in the occluded regions.

In [3], excavation changes were tracked by utilizing the depth difference in the terrain. This approach used laser scanners to track the terrain changes from two different locations that include the corner of a building and a steel pole located around the construction site. The point clouds from the two locations were then aligned using the registration technique. However, obtaining the truth reference ground is challenging due to the data noise and scan quality.

The excavated ground volume estimation sometimes faces the issue of unperceived areas due to the presence of obstacles such as a pile that blocks sensor vision and degrades the estimation accuracy [4]. Therefore, the necessity for another ground volume estimation is raised to avoid the occlusion problem. For example, the bucket capacity can be an indirect proxy for estimating the excavated ground volume. This is because it is possible to detect the soil volume in the bucket for the whole excavation period by mounting a sensor at the proper location near the bucket that can guarantee full visibility. 

There has been little effort to deal with the problem of occlusion areas occurring during the excavation. Some solutions to this problem were proposed in the legged robotics where the robot navigates under different terrains. Ref. [5] dealt with occluded rugged terrains due to the line-of-sight constraints of LIDAR sensors by applying the point-cloud matching and non-parametric terrain modeling algorithms. Through this method, the missing and occluded portions of terrains were filled in the height map. Another study [6] fused exteroceptive (data from a depth camera) and proprioceptive (foothold position of a legged robot) sensing to estimate the support surface for legged robots in vegetated (e.g., grass) environments that can obstruct the sensor visibility. This approach allowed fusing the foothold position variation and the vegetation height one, and creating the 2.5D height map as a representation of the estimated support surface.

An idea of applying the deep neural network to deal with the sensing occlusion has been proposed in [7]. This study utilized a LiDAR sensor to construct the lateral localization system. During the localization, detection of invisible (occluded) road boundaries was out by applying the occluded road boundary inference-deep neural network. For occlusion detection in the vehicle perception with LiDAR sensors, Ref. [8] developed a convolutional neural network (CNN)-based occupancy grid maps. Their approach can predict the locations of occluded objects emerging while overtaking in highway. Although the aforementioned methodologies could improve the quality and robustness of environmental monitoring by proposing the occlusion detection methods, their applications are limited to traffic and autonomous vehicles. Therefore, an occlusion detection strategy specialized for excavation applications needs to be explored.

Sensor fusion techniques have been adopted to recognize the motion and posture of construction equipment. In the study of [9] that developed an unmanned excavator to carry out dangerous construction works, various sensors including a GPS sensor, a potentiometer, and a tilt sensor were used to identify the excavator’s gripper position. Ref. [10] proposed a sensor fusion method by configuring both kinematic and audio signals that permits the classification of the construction equipment’s activities.

The commonly used sensor combination for 3D navigation and estimation of the ground surface in excavators includes the inertial measurement unit (IMU) fused with GPS or GNSS. Using the joint angle data obtained from this configuration and kinematic information, the dynamic status of an excavator was estimated, which can be further utilized for the estimation of a 3D profile of ground surface [11,12]. The stereo vision (depth camera)-based methodology has been utilized to identify the ground surface including rock piles [13] through stereo matching in the left and right images that allows obtaining the depth images of the surface. Although the stereo matching technique provides high-resolution depth information, its measurement accuracy is affected by the richness in the surface texture [14,15]. Despite the unique merits of these methodologies, no other info rather than the ground profile and distribution can be offered by them.

To tackle this limitation, the idea of a 4D map was proposed in [12] to identify the material properties. This map includes several components such as 3D geographic map and reflection data using a laser beam. It can provide extensive ground information by identifying material types using the reflectivity data. However, the method is not able to capture further information of the material properties like soil reaction force that can be considered as an important factor to carry out successful autonomous excavation. Therefore, an approach to incorporate the info of various material properties into a normal 3D map would be useful for monitoring and control of the excavation tasks.

By reflecting the critical issues in the above literature review, this paper presents three different aspects of the excavation progress monitoring, namely, excavated soil volume estimation, a solution to sensing occlusion, and 5D mapping for identifying the excavated material properties.

The excavation volume estimation is the first step towards the excavation progress that describes how much excavation has progressed in terms of dug volume. Two different estimation methods were investigated for this task, which include the direct estimation using the ground surface change and the indirect estimation through the soil volume contained in the bucket.

The second step towards the excavation progress is to overcome a technical challenge in ground mapping caused by the sensor occlusion problem. Specifically, surrounding obstacles and piles on excavation sites may block the view of sensors and result in limited environmental monitoring. To overcome this issue, we adopted the idea of fusing proprioceptive and exteroceptive measurements to reconstruct a ground map for the occluded areas.

The third focus of this paper is to generate the 5D map, which is referred to as a map that consists of a 3D ground map, the LiDAR intensity info (1D), and a force index (1D). The combination of all these entities can provide a wide spectrum of info on the excavated ground. To enhance the quality of the ground info produced from the 5D map, we also applied the convolutional neural network (CNN) to classify soil types using the ground images. Compared to the normal 3D or 4D map, the proposed 5D map can provide detailed information of the excavated ground that allows more accurate progress monitoring, and therefore sophisticated control of autonomous excavation based on that.

The remainder of this paper is outlined as follows. Section 2 provides an overview of the methodologies applied for this study. Section 3 describes the volume estimation algorithms. Section 4 explains the ground mapping in occlusion areas. In Section 5, the developed 5D mapping is described. Section 6 provides the results of experiments to validate the ideas proposed in Section 3, Section 4 and Section 5. Finally, Section 7 offers concluding remarks and suggestions.

## 2. Overview of Framework

**Figure 1:**
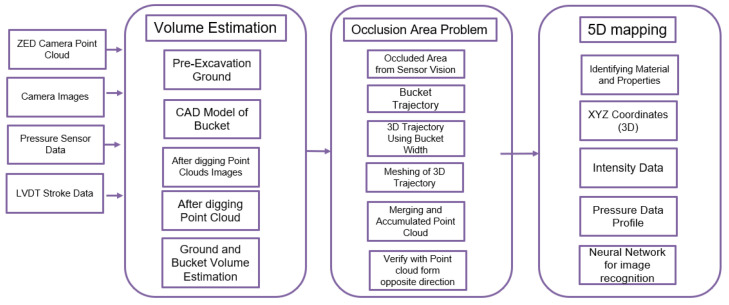
Integrative solution for monitoring autonomous excavation progress.

The primary goal of this paper is to propose an integrative solution for monitoring autonomous excavation progress using multiple sensors that can combine the critical components of ground volume estimation, map reconstruction for occlusion areas, and comprehensive ground mapping (5D map) for material property identification. Figure 1 shows a framework of this paper to achieve our objectives, which is composed of the aforementioned components. A detailed description of the framework is provided as follows.

The first part of the framework proposes the direct excavation volume estimation by applying the registration technique to align point clouds and the volume estimation technique (rasterization and height grids) to estimate the volume of the excavated ground. The first part also handles an alternative method for volume estimation. This method allows indirect monitoring of the excavation progress through estimating the volume of soil contained in the bucket that can be achieved by applying the registration technique to align the point cloud converted from the bucket CAD (Computer-Aided Design) model and the measured point cloud of the bucket with excavated soil.

The second part copes with the occlusion issue that is the major challenge to monitor the excavation progress or ground mapping. During the excavation, the sensor vision could often get blocked by a pile on the ground. As a solution to this problem, the bucket tip’s 3D trajectory was merged into a point cloud-based ground map for visible areas, which can fill the ground info of occluded areas.

Finally, the last part provides the info of the ground’s geometric space and material properties by generating the 5 D map that comprises the *x*, *y*, *z* coordinates, intensity value (LiDAR’s beam reflectivity), and a force index based on the actuator’s pressure data. To support the 5D map, the convolutional neural network technique was applied to classify the images that identify material types. The proposed integrative methodologies were developed in the MATLAB environment. A detailed explanation will be provided in the following sections.

## 3. Volume Estimation Algorithms

### 3.1. Excavated Ground Volume Estimation (Direct Estimation)

This section describes the developed algorithms for directly estimating the volume of the excavated ground. This approach is based on the volume calculation of dug areas that allows us to identify how much excavation is progressed. The direct estimation can be achieved by comparing the reference ground profile (reference point cloud) and the actual one (current point cloud) after excavation. The detailed procedure for this method is given as follows.

First, the reference point cloud that represents the pre-excavation ground profile was captured with the help of a stereo vision camera before the excavation. This reference point cloud was compared with the changes in the ground profile captured after each dig. To capture the current ground surface at each dig, the stereo camera was installed in a tilted position. Then, transformation matrices were applied to both point clouds (reference and current) to compensate for a tilting angle. After transforming the point clouds, the next step was to register these point clouds indicating the same scene and to integrate them into a common global coordinate system. Finally, the current point cloud was subtracted from the reference one. This subtraction means the volume difference between two point clouds (reference and actual ground profiles), which provides the info of the accumulated excavated volume.

### 3.2. Bucket Volume Estimation (Indirect Estimation)

The bucket volume estimation is a way to indirectly estimate the volume of the excavated ground during digging tasks. The bucket volume estimation is an effective method to monitor the excavation progress since the soil volume contained in the bucket can be detected by a sensor mounted on the excavator all the time during the entire excavation process. Hence, this method can overcome the main limitation of the direct estimation in Section 3.1 that it cannot accurately estimate the excavated ground volume if there is an occlusion area in digging space to hinder the detection of the ground profile.

As the first process of this method, the point cloud to represent the scanned bucket that contains the soil was captured using a stereo vision camera. The sensor position was fixed to the location that permits to fully capture the bucket volume during the excavation process. Then, the bucket CAD model was introduced to have the reference point cloud [16]. The registration technique was applied to align the point cloud of the scanned bucket with the reference point cloud. To fill out some vacant spaces in the point cloud, the triangulation meshing technique was adopted. Finally, the difference between the two point clouds (bucket containing the soil and bucket CAD model) was identified to compute the excavated volume after each dig.

## 4. Ground Mapping in Occlusion Areas

Another technical challenge in the ground mapping and autonomous excavation progress estimation comes from the occlusion areas that cause the limited or blocked Field of View (FOV) of sensors. Specifically, piles and other surrounding obstacles on the ground at excavation sites may limit the FOV of the sensors by blocking their vision and result in limited monitoring of the environment and excavation progress. As a remedy to the occlusion problem, we applied the method of fusing proprioceptive and exteroceptive sensors to reconstruct a map [4]. The proprioceptive sensors measure the internal dynamics of the excavator such as joint angles and body’s rotational position while the exteroceptive sensors gather the information from the external environment, i.e., distance measurement and ground surface profile.

### 4.1. Sensor Vision Occlusion and Initial Exteroceptive Map

**Figure 2:**
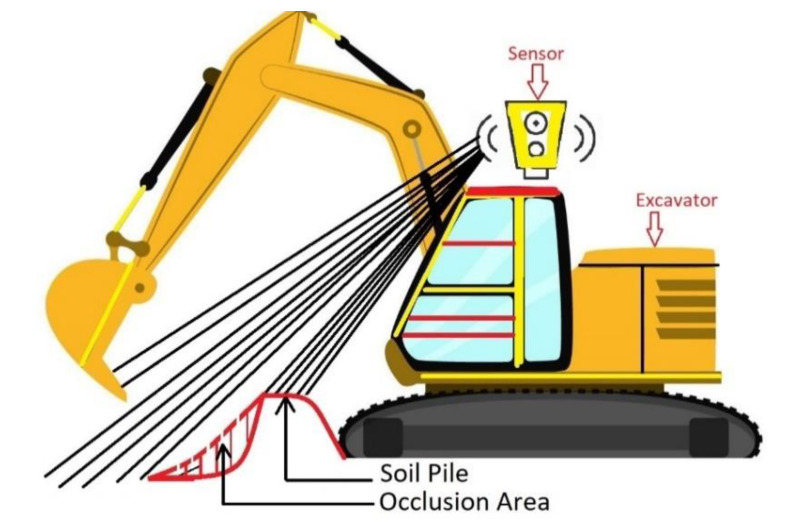
An example of an occlusion area: A pile on the ground blocks the sensor vision that hinders detection of the occluded area.

Figure 2 illustrates the sensor vision occlusion created by a dug pile with an excavator. The pile in front of the excavator blocks the vision of a sensor. In this case, the sensor is not capable of perceiving the ground on the other side of the pile where the vision is blocked (shaded area in the figure). Therefore, the exteroceptive sensor cannot collect the information on the ground surface in the occluded area. Instead, this study alternatively used a bucket trajectory as proprioceptive sensory data to get the geo-info of the occluded area that is always available regardless of vision blockage. Finally, the exteroceptive and proprioceptive data were integrated to monitor both unoccluded and occluded areas of the excavation site.

**Figure 3:**
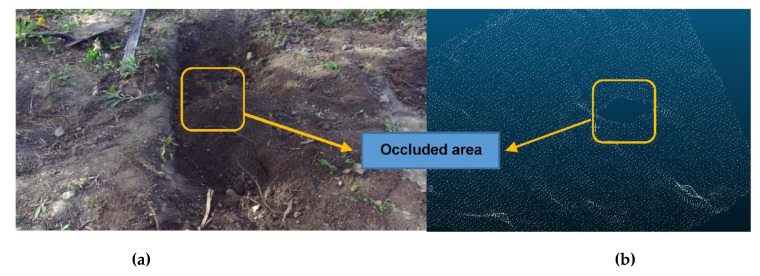
Example of an occluded region on the excavation site: (**a**) Indication of a pile that blocks the sensor vision causing occlusion; (**b**) Initial exteroceptive map using LiDAR sensory data (point cloud) for the same site that includes the identical occlusion region.

Figure 3b shows an initial map of the ground having an occlusion area that was constructed using a stereo vision camera (exteroceptive). As seen in the figure, the occluded area is included in the middle of the excavated ground. Thus, the initial map has the corresponding unscanned region where the vision of the camera is obstructed. To add the info of the ground surface in this region into the initial map, the bucket trajectory-based proprioceptive map was also created, which will be explained in the next section.

### 4.2. Identification of a Bucket Trajectory Using Kinematic Analysis

**Figure 4:**
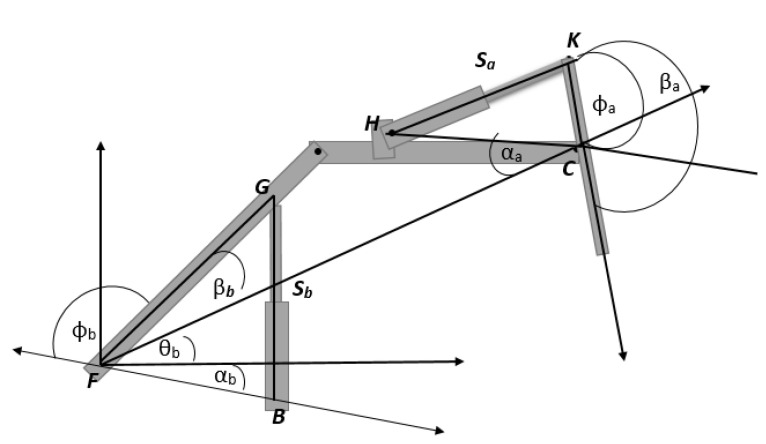
Joint angles and links for the excavator.

Tracking of a bucket trajectory during the digging task enables constructing the proprioceptive map for an occluded area. The first step in this mapping is to identify the trajectory of the bucket tip by using each actuator’s stroke data. The stroke data obtained from LVDT (Linear Variable Displacement Transducer) sensors were converted to each joint angle, and the position of the bucket tip was identified based on a kinematic analysis using the joint angles. The corresponding angles of each joint are shown in Figure 4 where $\theta_{b}$, $\theta_{a}$, and $\theta_{k}$ are the boom, arm, and bucket joint angles, respectively. The length information of the links presented in the figure is shown in Table 1.

#### Conversion of Stroke to Bucket Position

The boom angle can be calculated using the following equations.

(1) $$\emptyset_{b}=acos\left(\frac{{\bar{FB}}^{2}+{\bar{FG}}^{2}-S_{b}^{2}}{2\cdot \bar{FB}\cdot \bar{FG}}\right)$$

(2) $$\theta_{b}\;=\;\pi \;-\;\beta_{b}-\alpha_{b}-\emptyset_{b}.$$

The arm angle $\theta_{a}$ is obtained as follows:

(3) $$\emptyset_{a}=acos\left(\frac{S_{a}^{2}\;-\;{\bar{HC}}^{2}-{\bar{CK}}^{2}}{2\cdot \bar{HC}\cdot \bar{CK}}\right)$$

(4) $$\theta_{a}=\;2\pi \;-\;\beta_{a}-\alpha_{a}-\emptyset_{a}$$

**Figure 5:**
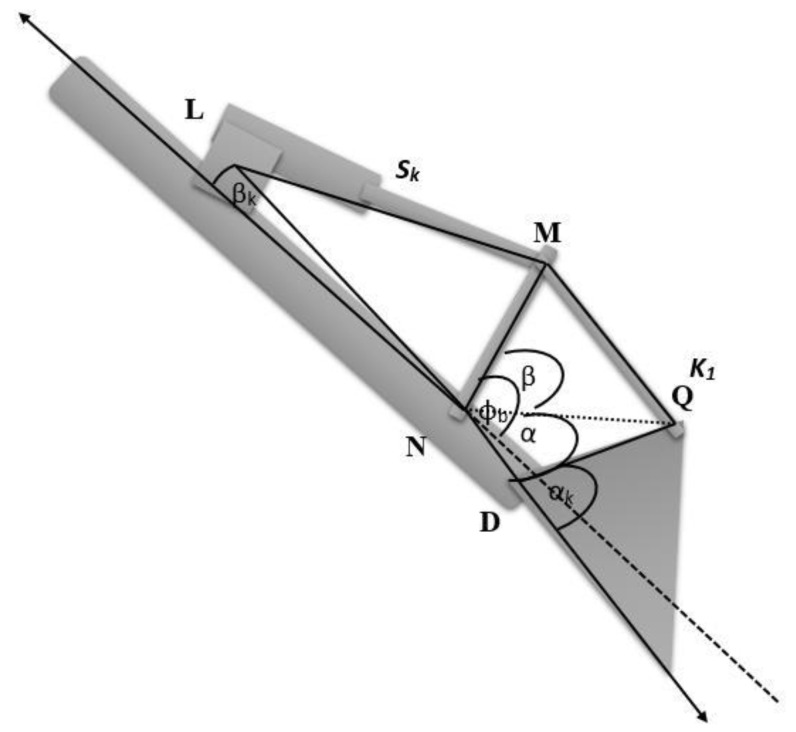
Bucket actuator and corresponding four-bar mechanism.

The bucket motion is driven by the four-bar mechanism NMQD (See Figure 5). The bucket angle $\theta_{k}$ can be calculated using the bucket stroke $S_{k}$ in the equation below:

(5) $$\emptyset_{k}=acos\left(\frac{{\bar{LN}}^{2}+{\bar{NM}}^{2}-S_{b}^{2}}{2\cdot \bar{LN}\cdot \bar{NM}}\right)$$

(6) $$MND\;=\;\pi \;-\;\beta_{4}-\emptyset_{k}$$

(7) $$k_{1}\;=\;\sqrt{{\bar{NP}}^{2}+{\bar{ND}}^{2}-2\cdot \bar{ND}\cdot \bar{NP}\cdot cos\left(\bar{MND}\right)}$$

(8) $$\alpha =acos\left(\frac{{\bar{ND}}^{2}+k_{1}{}^{2}-{\bar{NP}}^{2}}{2\cdot \bar{FB}\cdot k_{1}}\right)$$

(9) $$\beta =acos\left(\frac{k_{1}{}^{2}+{\bar{DQ}}^{2}-\;{\bar{PQ}}^{2}}{2\cdot \bar{DQ}\cdot k_{1}}\right)$$

(10) μ = α + β

(11) $$\theta_{k}\;=\;3\pi \;-\;\mu -\alpha_{k}$$

After computing the angles for each joint, the next step is to generate x and y coordinates of the bucket trajectory using these angles. The coordinates were calculated using the cosine law below.

(12) $$BucketTrackX\;=\;L2\cdot cos\theta_{b}\;+\;L3\cdot cos\left(\theta_{b}+\theta_{a}\right)\;+\;L4\cdot cos\left(\theta_{b}+\theta_{a}+\theta_{k}\right)$$

(13) $$BucketTrackY\;=\;L2\cdot sin\theta_{b}\;+\;L3\cdot sin\left(\theta_{b}+\theta_{a}\right)\;+\;L4\cdot sin\left(\theta_{b}+\theta_{a}+\theta_{k}\right)$$

where BucketTrackX and BucketTrackY are the x and y coordinates of the bucket trajectory.

**Figure 6:**
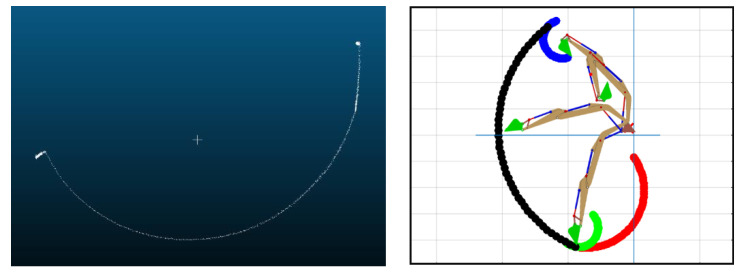
2D bucket trajectory.

**Figure 7:**
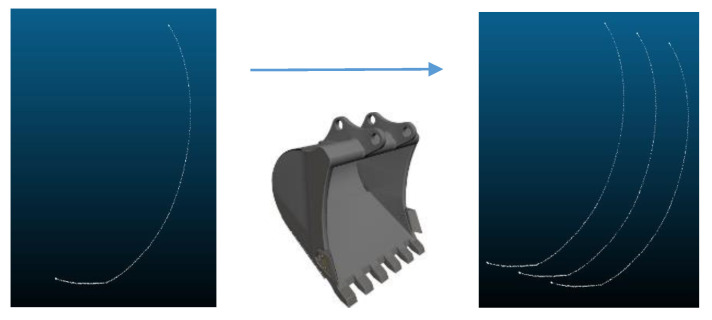
Conversion of 2D bucket path to 3D spatial trajectory.

Figure 6 shows an example of the bucket trajectory obtained after all the above computations. Since the above equations can generate a 2D bucket trajectory to identify the bucket tip’s path, it needs to be converted to 3D to reconstruct a ground map. For this 3D conversion, we considered the width of the bucket whose info with the 2D path can generate a 3D spatial trajectory. Figure 7 shows the converted bucket trajectory in 3D space. In the figure, the middle line shows the identified trajectory with regard to the manipulator’s centerline, and the other two lines present each outer side of the bucket (so, the distance between these two lines indicates the bucket’s width).

**Figure 8:**
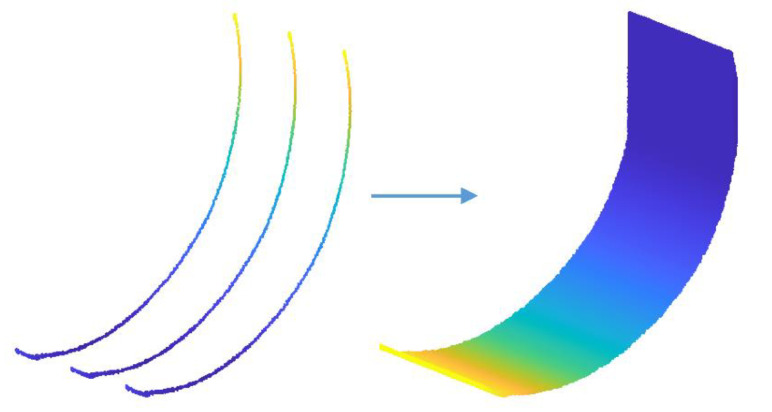
Application of the meshing technique to 2D bucket trajectory.

To fill the vacant spaces between these lines, we applied the meshing technique that is used for building a representation of 3D surfaces as a series of discrete facets [17]. Figure 8 shows the result of an application of the meshing technique to the 2D bucket trajectory.

### 4.3. Map Reconstruction for Occlusion Areas

**Figure 9:**
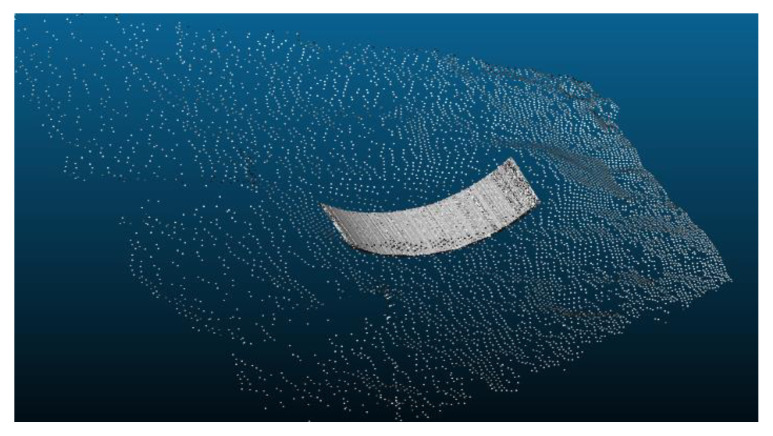
The final reconstruction map for occluded areas using proprioceptive and exteroceptive sensing data.

The reconstruction of a map for occlusion areas requires a fusion of proprioceptive and exteroceptive maps. For this process, a global coordinate system was defined first with its origin that was set to be the boom base of the excavator. Then, the sensor (local) coordinates were transformed into the global coordinate using the point cloud transformation matrices. After achieving a unified global coordinate system, the initial ground map built based on the vision sensor data was merged with a 3D mesh of the bucket trajectory. The final reconstructed map can be seen in Figure 9 where the occluded area in the initial map is filled with the proprioceptive map.

## 5. 5D Mapping

In this study, the 5D map is referred to as a map that consists of a 3D geometrical ground map, the intensity info based on LiDAR’s beam reflectivity (1D), and a ground force index (1D). The last two dimensions of the map are used to identify the excavated material properties and ground resistive force. Therefore, the 5D map can give a broad spectrum of the excavated ground info by combining all these entities. To enhance the functionality of this 5D map, we also applied the neural network (NN) technique to the ground images captured during the excavation for material classification.

### 5.1. 3D Geometrical Ground Map

The 3D geometrical map contains the geometric information of the excavated ground that includes the x,y, and z coordinates and shape. This info was obtained by using a stereo vision camera as it provides the dense point clouds and less vacant spaces between channels (laser breams). The registration technique was applied to build and accumulate the point clouds in a scene.

**Figure 10:**
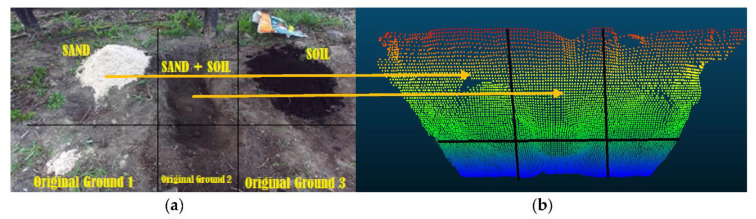
(**a**) Excavation ground segments considered for experiments; (**b**) Point clouds obtained from a stereo vision camera in each segment.

Among ICP [18], NDT [19], and CPD [20] as state-of-the-art registration techniques, NDT was chosen for this study by comparing each applied result in terms of the fast and robust registration. The excavation site for experiments is shown in Figure 10a that includes three segments with artificially supplied materials (sand, soil, and mixture) and three natural ground segments. Figure 10b presents the 3D map constructed using the point clouds from the camera in the considered six segments.

### 5.2. 3D Material Classification Using LiDAR’s Intensity

Although the stereo camera provides the geometrical data for a 3D map, this data is not sufficient to capture other crucial info of excavation materials that can be represented by the beam reflectivity or intensity [21]. To fuse the reflectivity info with the 3D map, we applied the intensity data from a LiDAR sensor as an index for the identification of material types [12]. This is because since the LiDAR can generate the intensity data used for numerous applications, such as wet area identification, land cover classification, distinguishing features, etc. [22]. However, we note that the intensity data alone is still not enough to classify the material as it cannot reflect the info of ground resistive force that occurs from the ground during digging. For example, the ground areas having the same surface reflectivity can show the identical intensity value but the depth or other ground conditions such as moisture content may result in a significant difference in ground resistive force. Therefore, we introduced the force index as an additional component of the 5D map that can be representative of the resistive force.

### 5.3. Indexing Ground Resistive Force

For the ground resistive force index of the 5D map, the net force of the bucket cylinder was considered. During the excavation cycle that consists of the penetration, dragging, and rotating (lifting-up), the bucket cylinder needs to be extended and generates the net force in Equation (14) acting against the ground resistance that can play the role of an index to represent the ground resistive force. As shown in Equation (14), the net force is calculated by subtracting the rod force from the head one using the pressure data (measured by pressure sensors attached to inlet and outlet ports of the bucket cylinder) and the areas of head and rod sides.

(14) $$F_{cyl}=P_{H}A_{H}-P_{R}A_{R}$$

where $F_{cyl}$ is the force exerted by the bucket actuator, $P_{H}$ is the head-side pressure, $\;A_{H}$ is the head area, $\;P_{R}$ is the rod-side pressure, and $A_{R}$ represents the rod area.

### 5.4. Soil Type Classification Using Convolutional Neural Network 

To strengthen our 5D map, neural network techniques were introduced to classify the types of soil images during the excavation process. To train the neural network, we adopted the idea of the transfer learning concept using MATLAB’s deep learning toolbox [23].

Transfer learning is a machine learning method where a pre-trained network (model) for a certain task can be utilized as a starting point for training another model with a different task. So, the learned features by this method can be transferred to a new task using a smaller number of data sets (images in our case). For the transfer learning, we chose the GoogleNet [24] network due to its less error rates and relatively faster training than other pre-trained networks [25]. The GoogleNet is a 22-layers deep network with the first input layer that requires the input images of size 224-by-224-by-3. Then, the convolutional neural network finalized from GoogleNet has 144 layers. Among the total data set of 130 images of soil and sand that were prepared for the neural network, 100 images were collected from Places365 (the latest subset of Places2 Database) online [26] and 30 images were taken during the experiments onsite. The entire data set was divided into the subsets for training (70%), validation (20%), and testing (10%).

## 6. Results and Discussions

### 6.1. Setup of a Test Platform

**Figure 11:**
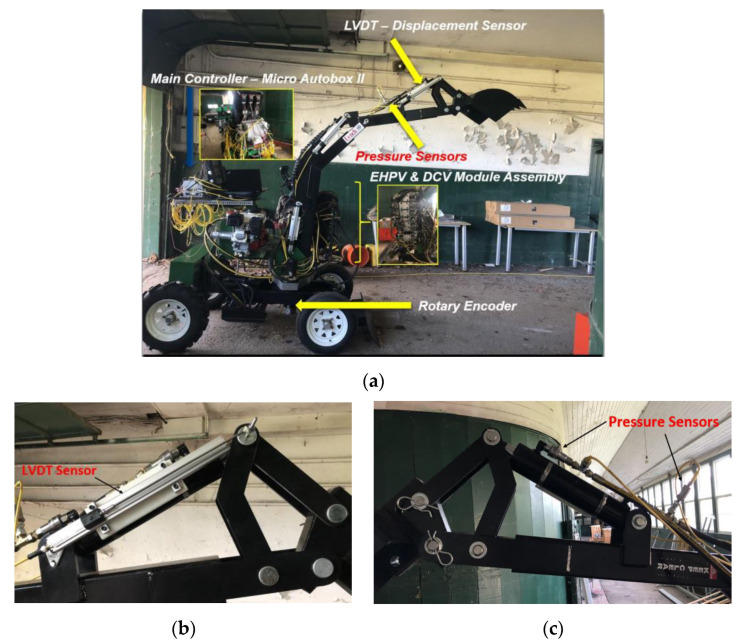
Test Platform Setup: (**a**) Modified mini excavator; (**b**) LVDT stroke sensor; (**c**) Pressure sensor.

The developed test platform is a modified mini hydraulic excavator (Figure 11a) that consists of three subsystems, i.e., electronic, hydraulic, and mechanical systems. The electronic system comprises electro-hydraulic drivers, a power supply, LVDT stroke sensors (Figure 11b), and pressure sensors (Figure 11c). The hydraulic system is composed of electro-hydraulic proportional valves, directional control valves, hydraulic actuators, and a hydraulic pump. The major mechanical system is a manipulator having three links for the boom, arm, and bucket, and joints.

As one of the sensor elements, a ZED stereo vision camera was selected. Since stereo vision has been widely adopted for 3D localization and detection of partially buried objects in the excavation systems, the ZED stereo vision camera was chosen to generate point clouds for the estimation of both ground and bucket volume. The position of the bucket was measured with the help of LVDT stroke sensors attached to each link of the excavator. The map reconstruction for occlusion areas was achieved based on the information of bucket trajectory. For 5D ground mapping, the info of geometrical coordinates was obtained using a stereo vision camera, and beam reflectivity was measured with a VLP-16 LiDAR. The force as the last component of the 5D map was calculated from the measurements of the pressure sensors attached to the cylinder inlet and outlet ports.

### 6.2. Estimation of Excavation Volume

Experimental results of ground excavation volume estimation and bucket volume estimation are presented and discussed in this section. To calculate the volume, the volumetric contribution of each cell (grid step/spacing) was summed up, which defines the volume of the parallelepiped elementary computed by multiplying the cell area by the difference of height [16].

#### 6.2.1. Estimation of Bucket Volume

**Figure 12:**
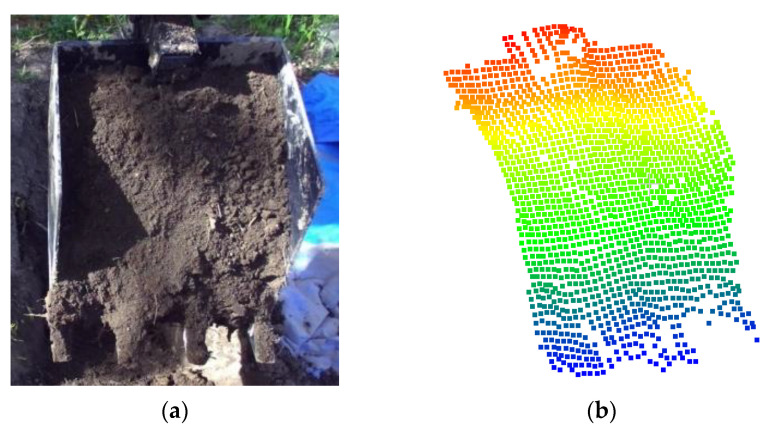
(**a**) Bucket volume image; (**b**) bucket point cloud.

The bucket volume is referred to as the soil volume that the bucket carries after each dig. Note that the bucket point cloud was captured after each dig during the excavation process for its volume estimation. As an example, the captured bucket volume’s image and the corresponding point cloud are provided in Figure 12a,b, respectively.

**Figure 13:**
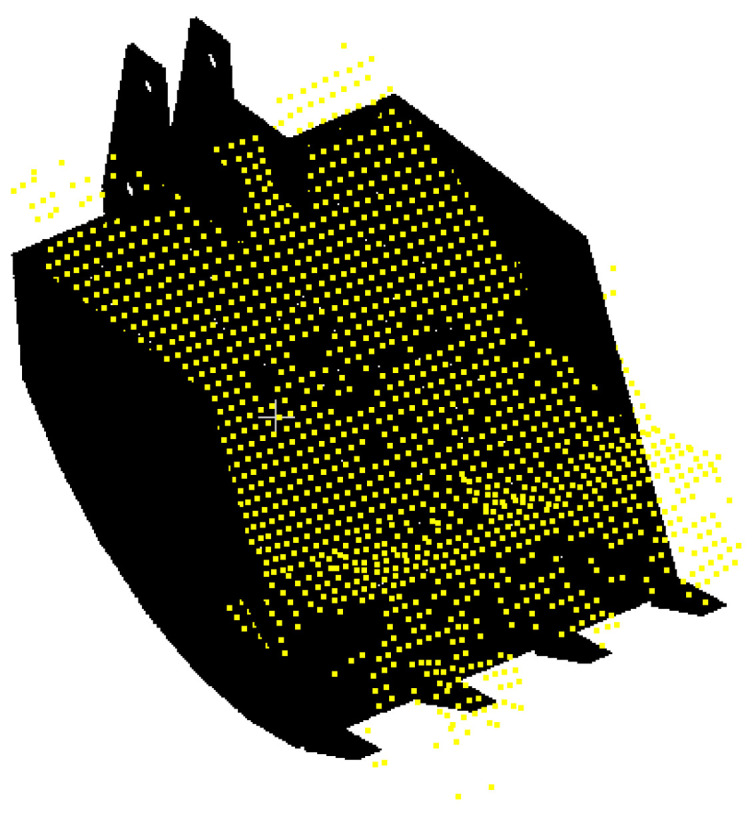
Merging the point cloud for the filled bucket soil volume into one for the CAD Model.

The point cloud presenting the soil volume contained in the bucket was merged with a CAD model after an application of the registration technique. Figure 13 illustrates the point cloud for the contained bucket volume that is aligned with one for the bucket CAD model.

**Figure 14:**
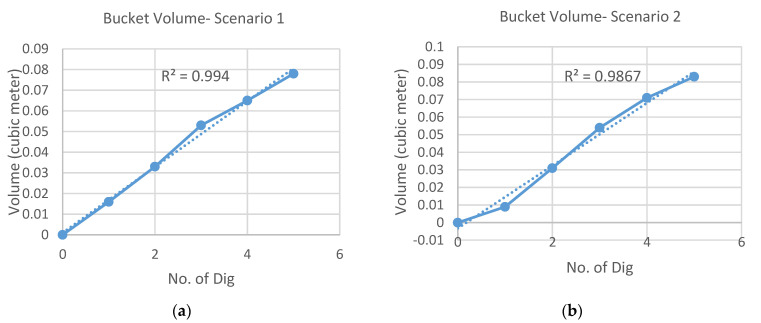
Results of bucket volume estimation: (**a**) Scenario 1; (**b**) Scenario 2.

For experimental validation, two different scenarios were considered. The first scenario (Figure 14a) keeps almost the same amount of incremental volume in each dig while the second scenario (Figure 14b) less uniform increment. As seen in Figure 14, five digs were carried out in both cases and the vertical axis shows the estimation of soil volume that is accumulated according to each dig.

#### 6.2.2. Estimation of Ground Excavation Volume

**Figure 15:**
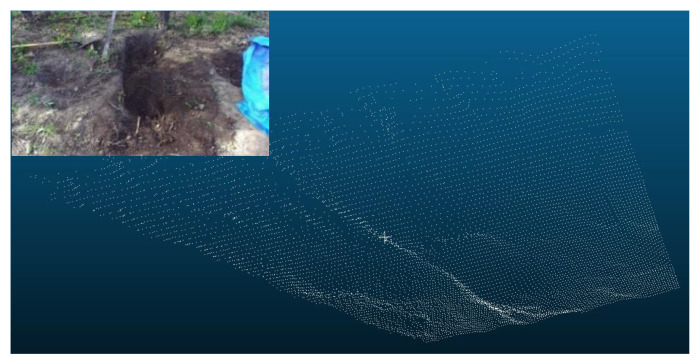
Point cloud of the ground surface before excavation.

**Figure 16:**
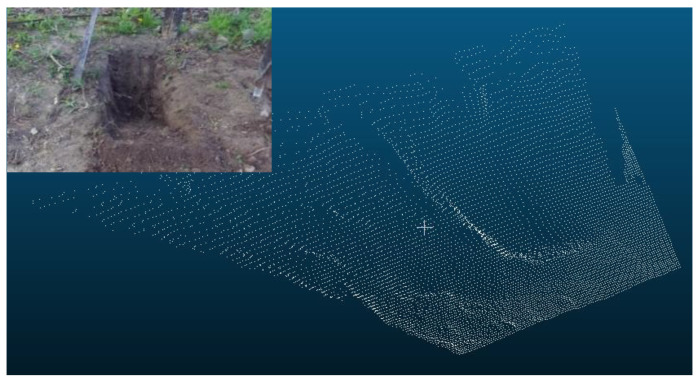
Point cloud of the ground surface after the 5th dig for the 1st scenario.

The ground excavation volume was estimated by calculating the difference (i.e., volume change) between the initial ground volume and the one after every dig using each measured point cloud that represents the ground surface profile. Figure 15 and Figure 16 show the point clouds of the ground before excavation and after the 5th dig for the 1st scenario as an example, respectively.

**Figure 17:**
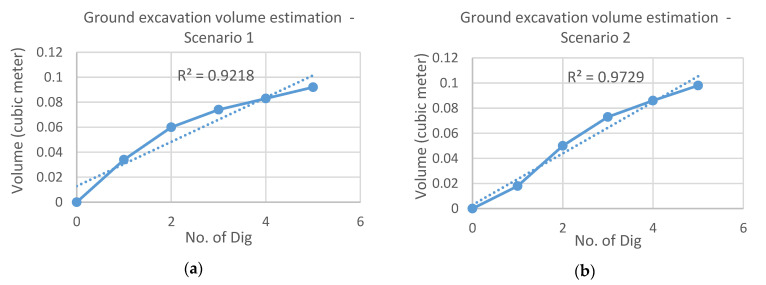
Result of ground volume estimation under Scenario 1 (**a**) and Scenario 2 (**b**).

The same scenarios considered in the bucket volume estimation were applied for the ground volume estimation. Below (Figure 17) are the two graphs that show the ground excavation volume estimation of each dig under the two scenarios.

#### 6.2.3. Relationship between Ground Volume and Bucket Volume

**Figure 18:**
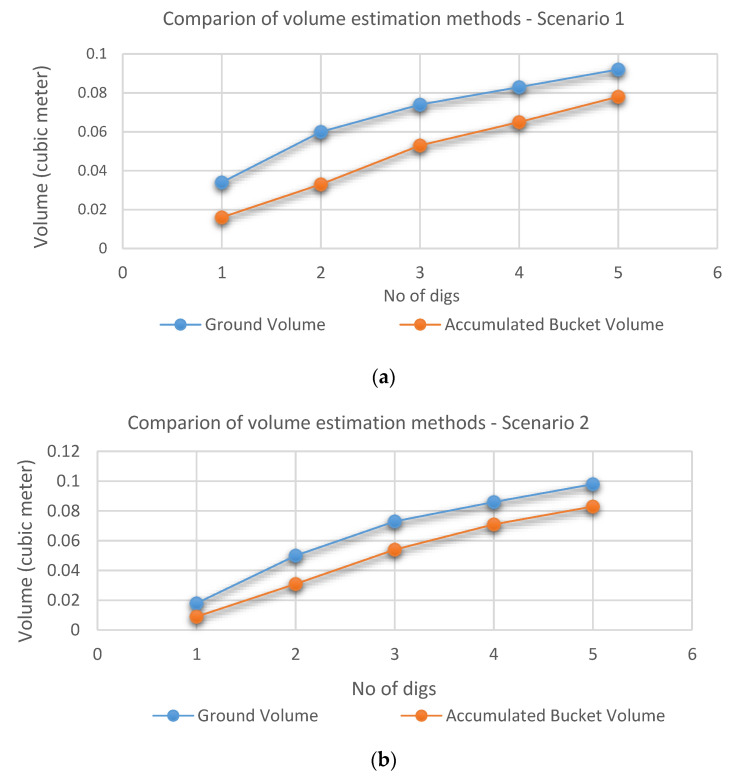
Comparison of the results of excavated volume estimation: (**a**) Scenario 1; (**b**) Scenario 2.

We compared the volume estimation results from each method for the same scenarios (Scenario 1 in Figure 18a and Scenario 2 in Figure 18b). In the figure, the blue and orange lines present the results obtained by the ground volume and accumulated bucket volume estimation methods, respectively.

From each sub-figure, we can see a similarity in graphs between both methods for each scenario. Therefore, this good correlation indicates that the bucket volume estimation can be used as an alternate way to estimate the ground volume to be dug during excavation progress. However, a constant offset between two graphs under each scenario is also observed. The reason for having such a difference can be explained as follows.

The stereo camera was used for both volume estimations, which provides dense point clouds. Then, we applied the registration technique where two point clouds to represent the same scene are matched as landmarks. Through this, the variation between frames can be checked. The ground volume estimation method has sufficient good landmarks from the 3D ground surface compared to the bucket volume estimation in which the number of landmarks is limited. Due to this reason, it is difficult to accurately identify the bucket’s edges using point clouds (despite their alignment with the bucket CAD model) and thus to distinguish between the point cloud from the bucket and the one from the contained soil at the edges. Hence, this difference affects estimation accuracy between the two estimation methods and results in a constant offset between two graphs as seen in Figure 18.

**Figure 19:**
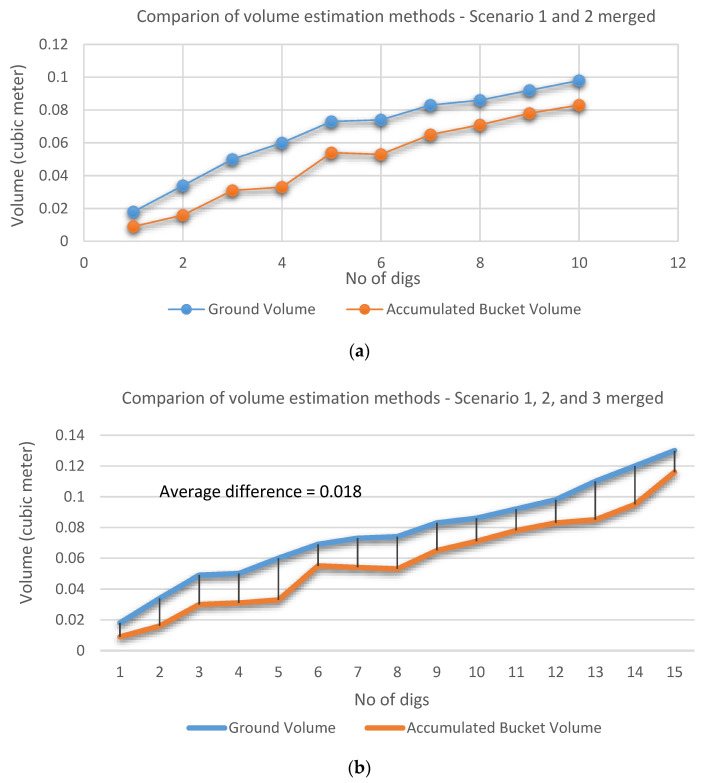
Merged data sets: (**a**) Scenarios 1 + 2; (**b**) Scenarios 1 + 2 + 3.

This correlation was further validated with the extended data sets in Figure 19b by adding the 3rd scenario data to the combined 1st and 2nd one in Figure 19a. The 3rd scenario consists of another five digs with an inconsistent volume increment.

**Figure 20:**
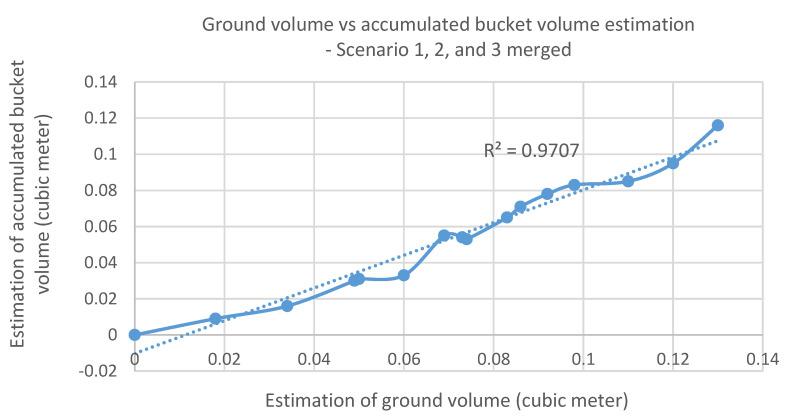
Relationship between the ground and accumulated bucket volume estimation.

Figure 20 shows the relationship between the estimated ground volume and the accumulated bucket volume obtained from the extended data sets (Figure 19b). From the graph, a strong positive linear relationship is observed except a few points. Table 2 shows the difference between these two volumes at each dig with the same extended data sets. Using the linear relation and the averaged difference or offset (0.018 m^3^) from the table, the ground volume during the excavation task can be alternatively estimated by the accumulated bucket soil volume. This approach can provide an effective solution to monitor the excavation progress through the indirect estimation of excavated volume at digging sites with occlusion areas since the bucket volume can be always monitored by a mounted sensor without having the occlusion issue. Another solution to deal with the occlusion areas will be introduced in the next section.

### 6.3. Mapping Using a Bucket Trajectory at Occlusion Areas

#### 6.3.1. Bucket Trajectory Formation

**Figure 21:**
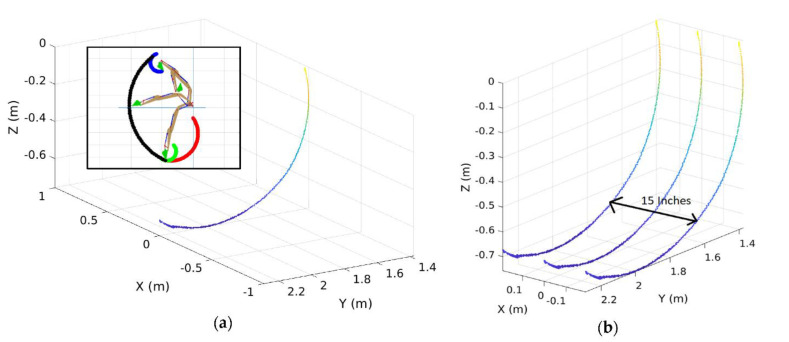
The identified bucket trajectory: (**a**) 2D bucket trajectory; (**b**) Converted 3D bucket trajectory by considering the bucket width.

To create a bucket trajectory, the stroke of the boom, arm, bucket actuators was measured by each corresponding LVDT sensor during the excavation time of 14 s with a sampling rate of 100 per second. Using Equations (1)–(11), the stroke can be converted to the joint angle. The position of the bucket tip was identified by applying the joint angle to the kinematic analysis in Equations (12) and (13). The obtained bucket trajectory through this procedure is shown in Figure 21. Then, this bucket trajectory was reconstructed to the 3D trajectory by considering the bucket width of 15 inches.

**Figure 22:**
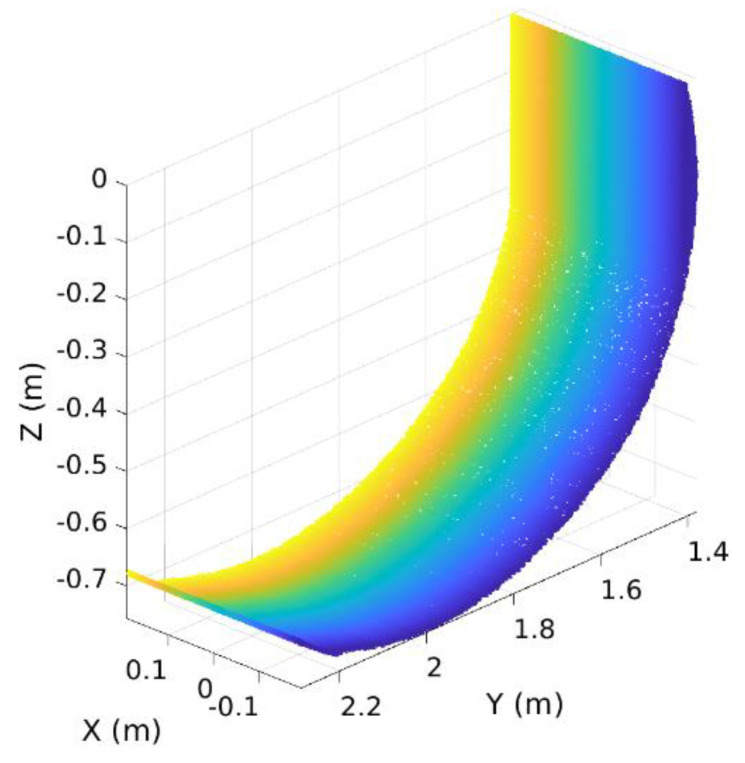
3D bucket trajectory after applying the triangulation meshing.

The next step is to fill out the vacant spaces between the 3D trajectory lines by using the triangulation meshing as shown in Figure 22.

#### 6.3.2. Map Reconstruction for Occluded Areas and Validation

**Figure 23:**
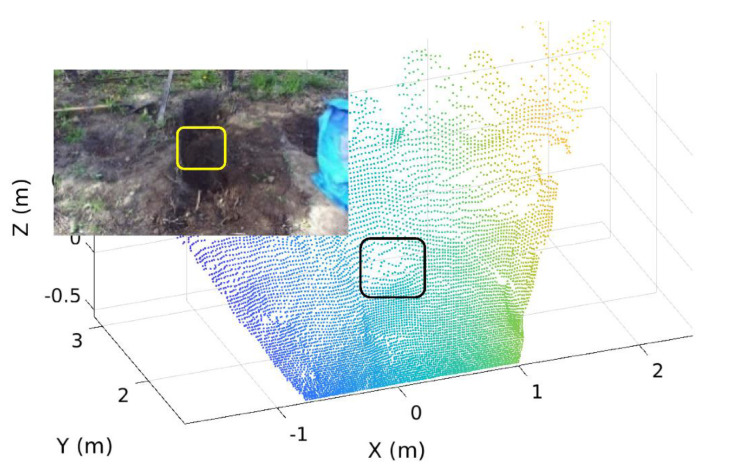
Occluded areas at the front side with the Field of View (FOV) blockage before excavation.

**Figure 24:**
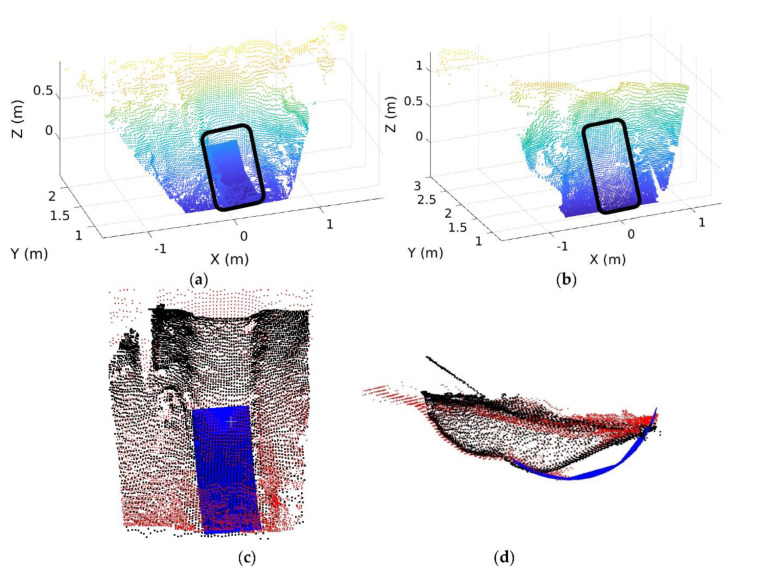
(**a**) Reconstructed map after excavation at the front side; (**b**) Ground map at the opposite side without the FOV blockage; (**c**) Overlap between Figure 24a,b in the top view; (**d**) Overlap between Figure 24a,b in the side view.

Figure 23 shows the occluded area occurring at the front side (i.e., original sensor location) before excavation for which the map reconstruction was considered in the study. Merging the 3D bucket trajectory (Figure 22) with the ground point clouds from the stereo vision sensor was carried to reconstruct a map for occluded areas, which is presented in Figure 24a that includes the blue surface as the 3D bucket trajectory. For this reconstruction, the coordinate systems for both (bucket trajectory and ground) point clouds were transformed into the pre-defined global coordinates.

The accuracy of the reconstructed map was validated by comparing it with the ground map using point clouds obtained from the same stereo vision camera installed on the opposite side (Figure 24b) where the same occasion areas can be detected without the FOV blockage.

By overlapping the point cloud in Figure 24a with the one in Figure 24b, the accuracy of the reconstructed map was analyzed as seen in Figure 24c,d where the red and black point clouds represent the reconstructed map (Figure 24a) and opposite side map (Figure 24b), respectively. The entire volume deviation between these two maps is 0.008 m^3^ in the overlapped map that is not significant. Therefore, it is confirmed that the reconstructed map with the bucket trajectory can be an alternative to the ground map that cannot provide the geo-info of occluded areas.

### 6.4. 5D Mapping

#### 6.4.1. 3D Ground Map and LiDAR’s Intensity

**Figure 25:**
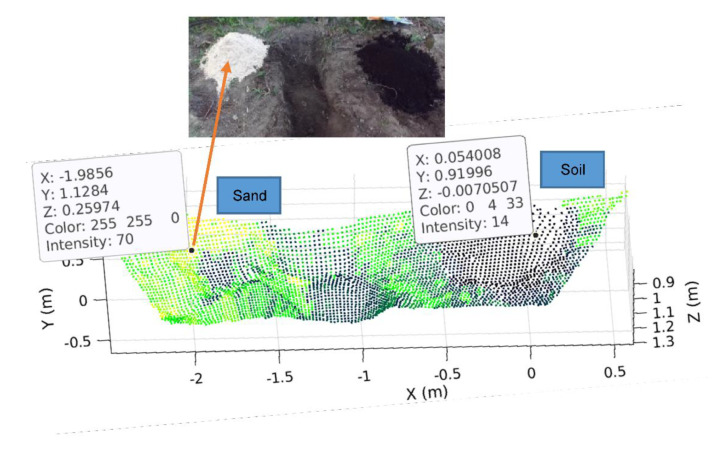
3D geometrical coordinates and intensity values for sand and soil.

The 5D map proposed in this study provides the ground info of 3D geometrical coordinates (i.e., 3D map), intensity, and force. Among them, the 3D geometrical coordinates and intensity were measured by a stereo camera and a LiDAR, respectively. Figure 25 shows a 3D map that shows the x, y, z coordinates of the ground targeted for excavation along with their corresponding intensity value obtained by a LiDAR.

In the figure, one can note that there is a significant difference in intensity between sand (70) and soil (14). Therefore, the intensity could be used as an index to identify the materials, but its value is subject to the feature of the detected ground surface. In particular, if the surface colors of two materials are similar, their intensity values are close. However, the information of the material (type) itself is not sufficient for the control of autonomous excavation that is also affected by the soil resistance arising from digging and dragging. To reflect this soil’s attribute on the ground mapping, we introduced the force index as an additional (5th) component that will be explained in the following section.

#### 6.4.2. Force Index

As the force index, the net force of the bucket cylinder was considered. This is because the net force of the bucket cylinder is used to be against the ground resistive forces during the excavation cycle that consists of the penetration, drag, and curl. For experimental validation of the force index, the net force was measured after the first dig in the target ground that has six segments in Figure 10 where the 1st, 2nd, and 3rd segments in the upper row were formed of sand, a mixture of sand and soil, and soil, respectively, and the rest three segments in the bottom row represent the natural ground. The segments in the upper row were artificially built by putting corresponding materials on the ground while the below three segments are the original ground surface.

**Figure 26:**
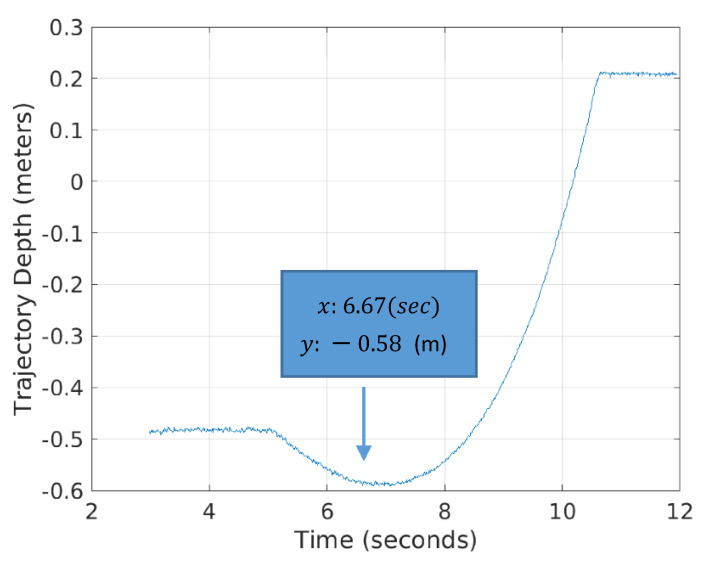
Bucket trajectory with respect to time during the excavation cycle at the 6th segment (Natural Ground 3).

To identify a digging point of the bucket on the ground surface during the excavation cycle, the bucket trajectory with respect to time was analyzed. Figure 26 shows the variation of the bucket trajectories in the 6th segment (Natural Ground 3) among six ones as an example. The lowest point in each trajectory presents the digging (contact) point of the bucket during the excavation cycle. After identifying the point of contact in time, the head force and rod force were calculated using the pressure data at the head and rod sides of the bucket cylinder and the areas of each side. The net force was calculated by subtracting the rod force from the head one.

**Figure 27:**
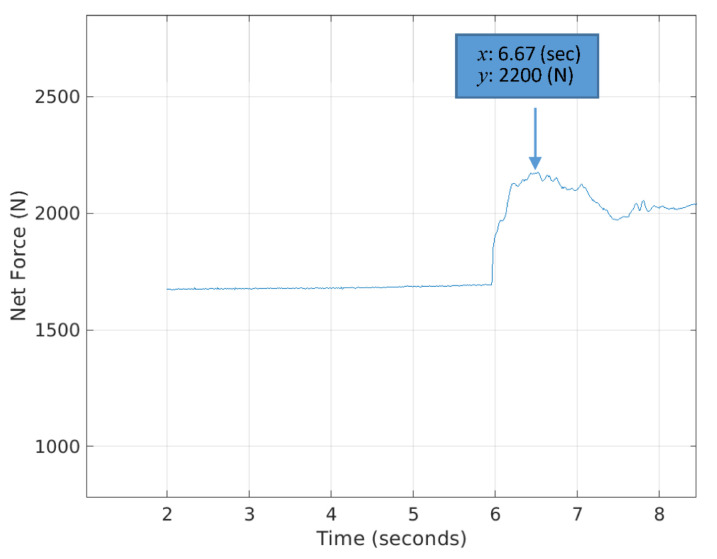
Variation of the bucket net force during the digging in the 6 segment (Natural Ground 3).

Note that Figure 27 illustrates the variation of bucket net force during the excavation cycle in the 6th segment and the contact point that coincides with 6.67 s in Figure 26. The value of net force at the contact point was selected as the force index for the 5D mapping.

#### 6.4.3. Relationship between Intensity and Net Force

The intensity from the LiDAR varies according to the color of the material surface (surface reflectance). For example, the intensity value becomes higher with sand than soil because sand has a brighter color (more reflective surface) and thus more energy can be reflected from its surface. The higher reflective surface of the sand than the soil can be confirmed by a higher intensity value in Table 3.

The 1st, 2nd, and 3rd segments (i.e., Segments 1, 2, and 3 in Table 3) were formed by artificially dumping the sand, mixture, and soil on the ground surface. Since these materials were not buried under the ground, the ground resistive force generated during the excavation in these segments is mostly lower than one in the natural ground (i.e., three segments in the bottom row in Figure 10) as seen in Table 3. Hence, the material property itself does not have a strong interdependence with the ground resistive force (or net force). Instead, the net force was investigated to see its relationship with the ground resistive force. Since actual digging occurs on the natural hard ground, such investigation was carried out with Segments 4, 5, and 6 in Table 3. Additionally, it is expected that the net force index can be associated with the digging depth. Therefore, we measured the digging depth for Segment 4, 5, and 6, which is ranked in the same order as the amount of the net force.

From the above results, we note that the net force can play a role of an index to indicate the resistive force arising from the ground contact that can be enhanced by an increase of the digging depth. Finally, since there is no regular pattern between intensity and net force for Segments 4, 5, and 6, the intensity is not suitable for an indicator to show the severity of the resistive ground force.

#### 6.4.4. 5D Map Construction

**Figure 28:**
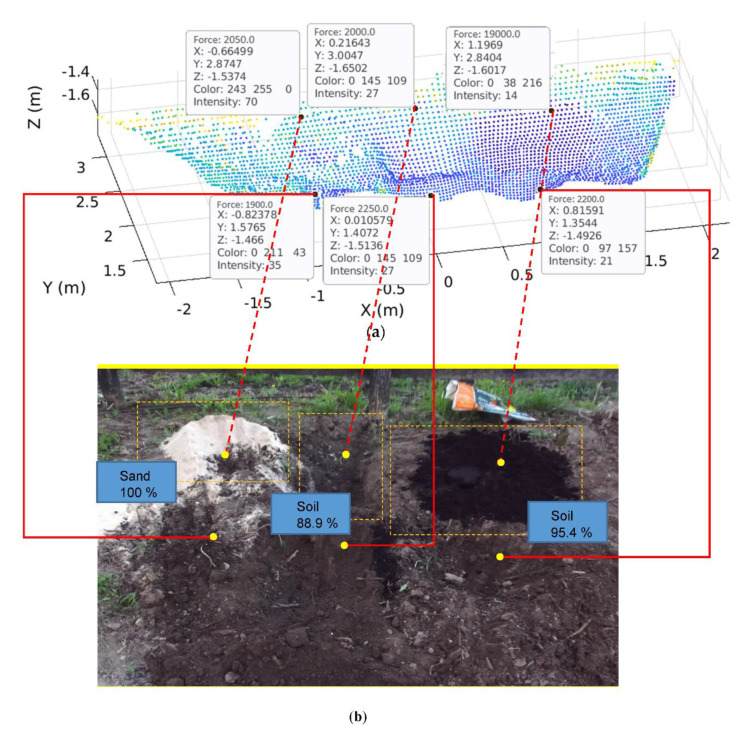
The constructed 5D map: (**a**) 5D point cloud map with respective material properties; (**b**) Actual photograph to show the monitoring target area.

The 5D map was constructed by integrating all the attributes of the 3D map, intensity, and net force. Figure 28a illustrates the 5D map to present the ground information of six segments having different types and properties of materials for the target area that is shown by the actual photograph of Figure 28b (equivalent to Figure 10a). The results of the intensity and force index addressed in Section 6.4.3 are included in this 5D map.

The image classification was also performed using the GoogleNet pre-trained classification network with the number of data sets described in Section 5.4. Through the classification results for the 1st, 2nd, and 3rd segments in Figure 28b (1st segment: Sand 100%; 2nd segment: Soil 88.9%; 3rd segment: Soil 95.4%), we can identify the corresponding soil type for each segment (i.e., pure sand, a mixture of sand and soil, and pure soil, respectively), and thus confirm that the trained neural network can successfully classify the types of excavation materials. These results match with the intensity magnitude (i.e., 70 (pure sand), 27 (mixture), 14 (pure soil)) obtained in Table 3.

Among three attributes of the 5D map used for monitoring the progress of excavations, the 3D map is used to update the current geo-information of the excavated terrain that is the critical information to track excavation progress and to generate the bucket tip’s path for the purpose of its position and trajectory control [27]. The intensity and force index provide a reference (desired) value for force tracking control that varies according to the ground conditions, and thus can be used to adaptively compensate for the ground resistive force. Therefore, the 5D map can fundamentally contribute to enhancing tracking control performance during digging operations [27,28].

## 7. Conclusions

This study proposes an effective integrative strategy required for excavation progress monitoring by dealing with the excavation volume estimation, occluded area, and 5D mapping. For the excavation volume estimation, two different methodologies, i.e., ground excavation volume estimation and bucket volume estimation, were studied to calculate the excavation progress.

The ground volume estimation as a direct estimation method was achieved by subtracting the actual ground profile after excavation from the reference one (pre-excavation ground surface). As an indirect approach, the soil volume contained in the bucket was estimated by aligning the point clouds for both the bucket CAD model and the bucket soil obtained using a stereo vision camera. From experimental validations, a strong positive relationship between the estimated ground volume and accumulated bucket volume was observed. Therefore, the bucket volume estimation method is applicable to monitor the excavation progress at digging sites with occlusion areas instead of the ground volume estimation method. This is because a camera can be mounted on the arm of the excavator, and thus the blockage problem of the camera view can be avoided during all the excavation process in the bucket volume estimation method.

To overcome the sensor occlusion problem due to a pile on the excavation ground, we considered an alternative approach to fuse the exteroceptive and proprioceptive sensors for ground mapping. The proprioceptive sensors (LVDT) helped in identifying the bucket trajectory while the exteroceptive sensor (stereo vision sensor) was used to build a ground map excluding the occlusion areas. The identified bucket trajectory was converted to 3D meshes using the triangulation method, which were finally merged with the exteroceptive ground map to reconstruct a complete 3D map.

In addition, a 5D map was created to provide the diverse excavation ground information that comprises a 3D map, the LiDAR intensity value, and the force index. Through this 5D map, the info of 3D geometrical coordinates, material type, and resistive ground force can be provided, which can facilitate accurate monitoring of excavation progress and control for autonomous excavation. Lastly, the convolutional neural network (CNN) was applied for the classification of soil types using the ground images taken during the excavation to enhance the 5D map’s functionality.

Although the proposed integrated strategy provides a unique solution to the problem of occlusion issue and the extensive ground information that is useful for monitoring and control of autonomous excavation beyond the current practice such as a normal 3D ground map, the following studies can be considered as future work to improve the quality of excavation progress monitoring

Since there has been a gap between the bucket volume estimation and ground volume one, a further study to reduce this gap through other registration techniques will be considered, which can enhance the estimation accuracy. In addition, validations on the exteroceptive and proprioceptive sensor fusion and the 5D map were carried out after a limited number of digs to check their feasibility. Therefore, additional tests under the ongoing and lengthy excavation progress are needed to ensure the practicality of these approaches.

The developed monitoring methodologies can be extensively used for other construction and agricultural equipment and off-road vehicles where environmental sensing and safety are critical to achieving autonomous operations.

## Figures and Tables

**Table 1:** Length and angle information of the links in Figure 4.

Measurement	Value	Measurement	Value
*FB*	0.175 m	*FG*	0.576 m
*HC*	0.549 m	*CK*	0.187 m
*LN*	0.450 m	*NM*	0.298 m
*PQ*	0.249 m	*DQ*	0.120 m
*ND*	0.111 m	$\beta_{b}$	31°
$\alpha_{b}$	45°	$\beta_{a}$	157.5°
$\alpha_{a}$	34°	$\beta_{k}$ *,* $\alpha_{k}$	15°, 87°

**Table 2:** Comparison of ground volume and accumulated bucket volume estimation.

No. of Digs	Ground Volume	Accumulated Bucket Volume	Difference (m^3^)
1	0.018	0.009	0.009
2	0.034	0.016	0.018
3	0.049	0.03	0.019
4	0.05	0.031	0.019
5	0.06	0.033	0.027
6	0.069	0.055	0.014
7	0.073	0.054	0.019
8	0.074	0.053	0.021
9	0.083	0.065	0.018
10	0.086	0.071	0.015
11	0.092	0.078	0.014
12	0.098	0.083	0.015
13	0.11	0.085	0.025
14	0.12	0.095	0.025
15	0.13	0.116	0.014
		Average	0.018

**Table 3:** Intensity and force index (net force) values for each segment.

Segment no and Type	Intensity	Force Index (Net Force) (N)	Digging Depth (m)
1 (Sand)	70	2050	Ground surface
2 (Sand + Soil)	27	2000	Ground surface
3 (Soil)	14	1900	Ground surface
4 (Natural Ground 1)	35	1900	0.55
5 (Natural Ground 2)	27	2250	0.65
6 (Natural Ground 3)	21	2200	0.58

## Data Availability

Data sharing not applicable.
